# Segmental spleen and left kidney infarction induced by vasoconstriction in a methamphetamine abuser patient: a case report

**DOI:** 10.1186/s12245-025-01011-1

**Published:** 2025-09-26

**Authors:** Alireza Javidan, Mahdi Rezai, Reza Mosaddegh, Najmeh Zarei Jelyani, Raha Latifaltojar

**Affiliations:** 1 Emergency Medicine Management Research Center, Health Management Research Institute, School of Medicine, Iran University of Medical Sciences, Tehran, Iran; 2 Department of Emergency Medicine, Faculty of Medicine, Shiraz University of Medical Sciences, Shiraz, IR Iran; 3 School of Medicine, Iran University of Medical Sciences, Tehran, Iran

**Keywords:** Methamphetamine, Vasoconstriction, Vasospasm, Infarction

## Abstract

**Background:**

Methamphetamine is one of the most used illicit drugs worldwide that affects different systems of the body by an immediate and sustained increase of monoamines. Its effect on the central nervous system (CNS) and neurotoxic effects are best known. There are also many reports of its effects on vasoconstriction in some peripheral and large caliber arteries, which results in different organ and limb disturbances.

**Case presentation:**

We report a rare case of a man with simultaneous spleen and left kidney infarction captured on contrast-enhanced computed tomographyeffects, without evidence of thrombosis or atherosclerosis, findings strongly suggestive of methamphetamine-induced vasospasm. The patient’s abdominal pain improved under observation within days without apparent tissue loss or organ failure. Unfortunately, the patient passed away as a result of bradycardia and cardiac arrest.

**Conclusion:**

This very rare case highlights the potentially severe vasospastic complications of methamphetamine abuse, which may result in multiorgan infarction. The fatal outcome in this patient was primarily due to underlying cardiac disease and endocarditis, although methamphetamine-associated cardiovascular strain could not be excluded as a contributor. Clinicians should consider vascular events in methamphetamine users presenting with atypical abdominal pain or any signs of organ dysfunction.

## Introduction

Methamphetamine is a kind of amphetamine-type stimulant that was first obtained from the structural change of ephedrine as a precursor.

The first medicinal use of methamphetamine dates back to the early 1930 s for its bronchodilator effects. Other therapeutic effects, such as treating schizophrenia, narcolepsy, addiction, hypotension, heart block, epilepsy, and some neuromuscular diseases, as well as weight loss, although its use has been largely discontinued because of its high potential for abuse and serious adverse effects [1, 2].

Nowadays, Amphetamines are the third most commonly used illicit drugs worldwide after cannabis and opioids [3].

The addictive properties of methamphetamine are related to the immediate and sustained increase of monoamines (dopamine, serotonin, and norepinephrine) by the blockade of presynaptic reuptake, which results in postsynaptic neuron hyperstimulation [4]. Beyond the well-known neurocognitive and psychiatric effects, including mood and excitation level alterations, psychotic behaviors and appetite alterations methamphetamine has also been associated with severe vascular complications [1, 2, 4]. Amphetamine and methamphetamine are known to cause intense vasoconstriction, which has been linked to ischemic stroke, myocardial infarction, intestinal ischemia, and peripheral vascular compromise [5–13].

Here, we present what appears to be the first reported case of concurrent splenic and left renal infarction attributable to methamphetamine-induced vasoconstriction, highlighting the importance of considering vascular events by clinicians in methamphetamine users presenting with abdominal pain.

## Case report

A male patient in mid-30s with a complaint of acute onset of left upper quadrant, left hemithorax and epigastric pain with one week of hematuria came to our emergency department.

He mentioned that his pain was not positional and started about one week ago, which was controlled with painkillers at first, but it worsened over time without any history of trauma or other symptoms.

The patient was afebrile with stable hemodynamics on admission and without any sign of intoxication.

On physical examination, the left upper quadrant of the abdomen was slightly tender without any guarding, rebound tenderness, or other significant signs and symptoms. The formed stool was identified in the digital rectal exam.

The history of the patient revealed pulmonary thromboembolism about two years ago, cardiac arrest, and following cardiopulmonary resuscitation about nine years before his admission.

His surgical history was liposuction surgery about 10 years ago and ureteral stent placement about 11 years ago.

Further questions about the patient’s social history revealed that he was a heavy smoker and consumed daily methamphetamine since five years ago with the last use occurring the day before symptom onset.

He had no drug history, except for the recent use of analgesics for his abdominal pain.

The following were his vital signs: temperature of 36.6 °C, pulse rate of 75 bpm, respiratory rate of 18, oxygen saturation of 97%, and blood pressure of 145/90 mmHg. The patient’s first laboratory findings are summarized in Table 1. A hypercoagulable workup was not pursued due to limited resources and the presence of an alternative plausible etiology.

**Table 1:** Summary of the patient’s laboratory test results

Test	Result	Reference Range	Unit
White Blood Cell	12.8	4.0–10.0	10^3^/mm^3^
Neutrophils (Segmented)	80	40–75	%
Lymphocyte	10	20–45	%
Mixed	7	2.0–10	%
Red Blood Cell	4.3	Male:4.5–6.3	10^6^/mm^3^
Hemoglobin	13.0	Male:14.0–18.0	g/dl
Platelet	163	140–440	1000/mm^3^
Blood Sugar	136	70–115	mg/dl
Blood Urea Nitrogen (BUN)	10	5.0–23.0	mg/dl
Creatinine	1.2	0.5–1.5	mg/dl
Creatine Phosphokinase (CPK)	279	24.0-195	IU/L
CPK-MB	29	0.0–30	U/L
Serum Sodium(Na)	134	136–145	mEq/L
Serum Potassium(K)	3.5	3.7–5.5	mEq/L
Lactate Dehydrogenase (LDH)	762	225–500	U/L
Alanine Aminotransferase (ALT)	59	5.0–40	IU/L
Aspartate Aminotransferase(AST)	61	5.0–40	IU/L
Alkaline Phosphatase (ALP)	130	64–306	IU/L
Bilirubin total	1.8	0.1–1.2	mg/dl
Bilirubin direct	0.5	0.0-0.4	mg/dl
Amylase	65	20.0-104	U/L
Lipase	18	<=60	U/L
Prothrombin Time (PT)	15.3	12–13	Sec.
International Normalized Ratio (INR)	1.18	1.0-1.1	Index
Partial Thromboplastin Time (PTT)	32	25–40	Sec.
Troponin - I	5.2	Normal: <19 Observation: 19–100 damage: >100	ng/L
Urine Toxicology (Qualitative)	Methadone (MTD): Negative Methamphetamine (MET): Positive Amphetamine (AMP): Positive Cannabis (THC): Negative		

Urine toxicology was also positive for methamphetamine and amphetamine. (Table 1) Upright chest X-ray and abdominal X-ray were initially done in the emergency department.

Based on the patient’s left hemithorax pain and suspicion of pulmonary embolism, the obtained CT Angiography revealed an increased diameter of the pulmonary trunk right ventricular (RV) strain suggestive of chronic thromboembolic pulmonary hypertension (CTEPH), along with bilateral patchy ground-glass opacities. No evidence of intraluminal filling defects suggestive of pulmonary artery thromboembolism was observed.

**Fig. 1:**
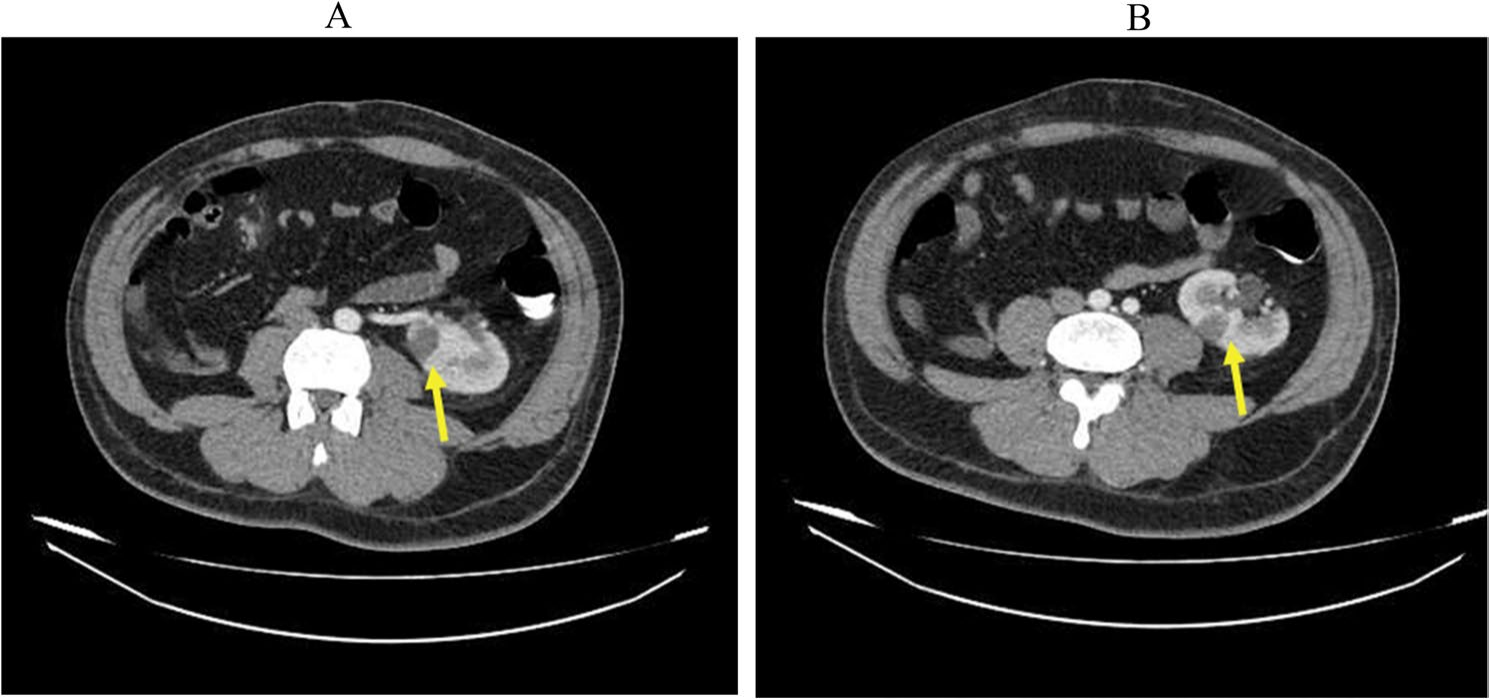
Axial Abdominopelvic CT with intravenous contrast. **A **Wedge-shaped hypoenhancement in the left kidney suggesting renal infarction. **B** Another slice from the same abdominal CT scan, demonstrating the same lesions. (Arrows indicate areas of infarction)

**Fig. 2:**
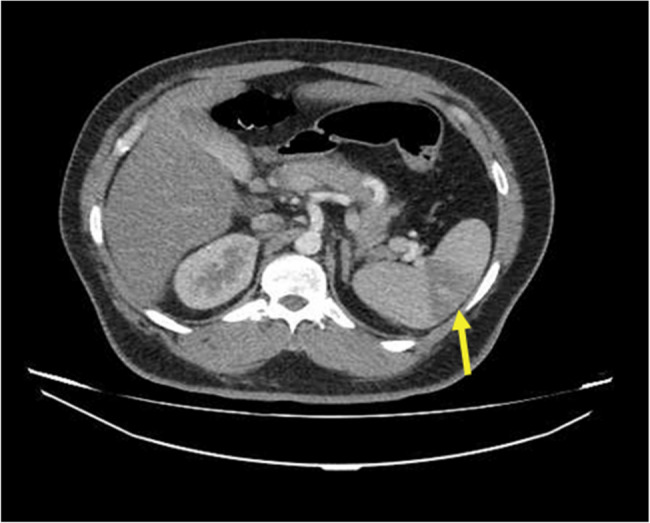
Axial Abdominopelvic CT with intravenous contrast. Splenic infarction visible as a wedge-shaped hypodense area in the spleen. (Arrow indicates area of infarction)

Based on multiple air-fluid levels, Abdominopelvic CT with intravenous contrast was obtained to rule out obstruction. This CT showed multiple wedge-shaped hypodense areas suggesting infarctions in the mid-splenic pole and the mid-zone of the left kidney without any sign of thrombosis and atherosclerotic changes in major abdominal vessels, which could result from arterial vasospasm (Figs. 1A and B, and 2).

Based on the initial concern for thromboembolic or thrombotic events, heparin therapy was initiated and continued during hospitalization. The patient’s abdominal pain was improved under observation within two days. During the admission, the patient’s echocardiography and transthoracic echocardiography showed aorta and pulmonary valve endocarditis and aortopulmonary fistula secondary to it with no evidence of thrombosis or atherosclerotic changes in the major abdominal vessels. He underwent cardiac surgery and later sternotomy as a result of subsequent cardiac tamponade. He also underwent hemodialysis after following acidosis and hyperkalemia and, unfortunately, died during hemodialysis most likely related to his underlying cardiac disease rather than direct methamphetamine toxicity.

## Discussion

Methamphetamine, an amphetamine derivative, is a CNS stimulant called variously as a “crystal”, “meth”, or “speed” and can be injected, smoked, snorted, or taken orally [14].

Methamphetamine was first synthesized in 1887, and its first medical use dates back to the early 1930 s to treat tiredness in fatigued army troops. It was widely used to treat narcolepsy, attention deficit hyperactivity disorder, depression, alcoholism, and to decrease appetite for weight loss [2, 14].

The structural similarity of methamphetamines to catecholamine transporters changes the endogenous function of these transporters, resulting in more release and decreased reuptake of catecholamines in the CNS. It also inhibits monoamine oxidase function, which results in the build-up of more catecholamines in the synapses [15].

Immediate effects of methamphetamine use include euphoric effects, arousal, cognitive and memory changes, increases in blood pressure, heart rate, body temperature, pain perception changes, and loss of appetite [15, 16].

The chronic use of methamphetamine results in changes in behavior and cognition, memory deficit, psychosis, violent behaviors, severe anxiety and depression, immune system issues, liver and kidney damage, respiratory issues, cardiovascular impairment as infarction, cardiomyopathy, and arrhythmias, gastrointestinal issues as a result of vasoconstriction and bowel ischemia, and immune system issues. Later complications are due to the prolonged and severe vasospasm after methamphetamine consumption [15–17].

Different studies show the impact of methamphetamines on the narrowing of blood vessels because of vasospasm, which leads to ischemic stroke due to cerebral vessel narrowing, ischemic colitis, and bowel ischemia due to mesenteric vasospasm, myocardial infarction, and extremities vasospasm [7, 18–21]. methamphetamine increases synaptic norepinephrine, leading to intense α-adrenergic–mediated vasoconstriction and prolonged vasospasm, which is the most plausible mechanism in our case [16]. 

Our case is unique in demonstrating simultaneous segmental infarction of the spleen and left kidney after methamphetamine use, without evidence of thrombosis or atherosclerotic changes in the imaging studies. While isolated stimulant-related renal infarction has been described (e.g., Khokhar et al., 2022) [22], to our knowledge this is the first report of concurrent renal and splenic infarction attributable to methamphetamine use.

As in most stimulant-associated vascular events, the diagnosis of methamphetamine-induced infarction was reached by exclusion. During hospitalization, given the differential consideration of septic embolism and embolic infarcts secondary to endocarditis, transthoracic and transesophageal echocardiography demonstrated endocarditis of the aortic and pulmonary valves with an aortopulmonary fistula. However, at admission the patient was afebrile, without evidence of infection, and no vegetations or embolic sources were identified at the time of splenic and renal infarction. In addition, there were no systemic inflammatory signs, abnormal serological markers, or vessel wall changes suggestive of vasculitis. Taken together with the CT findings of segmental organ infarction in the absence of thrombosis or atherosclerosis in major abdominal vessels, These findings, combined with the patient’s strong history of daily methamphetamine use, made vasospasm the most plausible explanation. Although a full hypercoagulable workup was not performed, no clinical or laboratory evidence supported an underlying thrombophilia, which we acknowledge as a limitation.

Heparin was initiated based on the patient’s initial clinical presentation and the suspicion of embolic or thrombotic events as a differential diagnosis, and conservative management with anticoagulation was therefore considered appropriate. The patient’s abdominal pain improved after heparin initiation, though a causal link cannot be confirmed, as spontaneous resolution of vasospasm may also be possible. In similar cases, earlier interventions targeting arterial vasospasm, such as vasodilators, could theoretically be beneficial, although evidence for their use in methamphetamine-associated organ infarction is limited.

Despite improvement in abdominal pain, the patient later developed infective endocarditis of the aortic and pulmonary valves complicated by aortopulmonary fistula and tamponade, requiring surgery and hemodialysis. He ultimately died of bradycardia and cardiac arrest during dialysis. Although the fatal outcome was primarily due to infective endocarditis and its complications, methamphetamine-associated vasospasm and cardiovascular strain could not be entirely excluded as potential contributors to the adverse outcome. Other limitations of this report include its single-case design, lack of autopsy to confirm the duration of vasospasm, lack of advanced investigations (such thrombophilia panels, or detailed inflammatory markers), and potential confounders such as prior cardiac disease and smoking, which may have contributed to the outcome. This highlights the multifactorial nature of morbidity and mortality in such patients. Future directions may include the potential role of angiography in confirming vasospasm, as well as routine vascular screening and earlier interventions, such as vasodilators, in methamphetamine users to identify and prevent early complications.

This case highlights that methamphetamine can cause multi-organ ischemia through vasospasm, and that severe outcomes, including death, may occur. Clinicians should therefore maintain a high index of suspicion and consider early vascular imaging in methamphetamine users presenting with unexplained abdominal pain or organ dysfunction. Given the extreme rarity of simultaneous splenic and renal infarction, early recognition and awareness are essential for improving outcomes.

## Conclusion

Methamphetamine is one of the most used illicit drugs worldwide, with well-known effects on the CNS. There are also many reports and studies about the effects of methamphetamine on the vasospasm of small and large vessels in different organs and limbs. Based on the severity and duration of reduced perfusion, it can have different debilitating consequences. clinicians should be aware of simultaneous infarctions in multiple organs due to arterial vasospasm, even in the absence of thrombosis in patients with a positive history of methamphetamine use. Urine drug screen tests and imaging studies are necessary to confirm the diagnosis. Careful monitoring along with early interventions may improve patient outcomes. the potential role of angiography in confirming vasospasm, as well as routine vascular screening and interventions, such as vasodilators, warrants further study.

## Data Availability

All data generated or analyzed during this study are included in this published article. Further information is available from the corresponding author upon reasonable request.
